# MiSurv: an Integrative Web Cloud Platform for User-Friendly Microbiome Data Analysis with Survival Responses

**DOI:** 10.1128/spectrum.05059-22

**Published:** 2023-04-11

**Authors:** Won Gu, Hyunwook Koh, Hyojung Jang, Byungho Lee, Byungkon Kang

**Affiliations:** a Department of Applied Mathematics and Statistics, The State University of New York, Korea, Incheon, South Korea; b Department of Computer Science, The State University of New York, Korea, Incheon, South Korea; American Type Culture Collection

**Keywords:** microbiome data analysis, web cloud platform, survival analysis, association analysis, prediction modeling

## Abstract

Investigators have studied the treatment effects on human health or disease, the treatment effects on human microbiome, and the roles of the microbiome on human health or disease. Especially, in a clinical trial, investigators commonly trace disease status over a lengthy period to survey the sequential disease progression for different treatment groups (e.g., treatment versus placebo, new treatment versus old treatment). Hence, disease responses are often available in the form of survival (i.e., time-to-event) responses stratified by treatment groups. While the recent web cloud platforms have enabled user-friendly microbiome data processing and analytics, there is currently no web cloud platform to analyze microbiome data with survival responses. Therefore, we introduce here an integrative web cloud platform, called MiSurv, for comprehensive microbiome data analysis with survival responses.

**IMPORTANCE** MiSurv consists of a data processing module and its following four data analytic modules: (i) Module 1: Comparative survival analysis between treatment groups, (ii) Module 2: Comparative analysis in microbial composition between treatment groups, (iii) Module 3: Association testing between microbial composition and survival responses, (iv) Module 4: Prediction modeling using microbial taxa on survival responses. We demonstrate its use through an example trial on the effects of antibiotic use on the survival rate against type 1 diabetes (T1D) onset and gut microbiome composition, respectively, and the effects of the gut microbiome on the survival rate against T1D onset. MiSurv is freely available on our web server (http://misurv.micloud.kr) or can alternatively run on the user’s local computer (https://github.com/wg99526/MiSurvGit).

## INTRODUCTION

The human microbiome is the entirety of the microorganisms that reside in and on the human body. The advances in high-throughput sequencing have greatly lowered the cost for microbiome profiling and improved the accuracy of the microbiome data. Then, investigators have found that the dysbiosis of the human microbiome is linked to numerous human diseases, for example, through studies on the effects of a medical treatment on human microbiome (1–6), the roles of the microbiome on human health or disease (1, 2, 4, 7–14), and so forth.

There have also been many analytic pipelines and tools that are developed for microbiome data processing and downstream data analysis. Some example microbiome profiling pipelines are QIIME (15), QIIME2 (16), MG-RAST (17), Mothur (18), and Nephele (19) for 16S rRNA sequencing, and MEGAN (20) and MetaPhlAn (21) for shotgun metagenomics. Some example downstream microbiome data analytic tools are aMiAD (22) for alpha-diversity analysis, PERMANOVA (23–25) and MiRKAT (26–28) for beta-diversity analysis, and metagenomeSeq (29) and ANCOM (30) for taxonomic differential abundance analysis. Among those, we especially pay attention to the web cloud platforms, such as Nephele (19), Qiita (31), PUMAA (32), MicrobiomeAnalyst (33), METAGENassist (34), EzBioCloud (35), and MiCloud (36). These web cloud platforms have had the advantages of (i) user-friendly operation, (ii) streamlined analytic pipeline, and (iii) cloud computing service over the past software packages based on command-line interfaces. Thereby, investigators can easily deal with highly complex microbiome data, with no professional programming skills, not spending too much time and effort for a long sequence of data processing and analytic tasks, and with no fancy computer to process a huge amount of information.

However, for downstream microbiome data analysis, there is currently no web cloud platform available to analyze microbiome data with survival (i.e., time-to-event) responses. Investigators have been conducting numerous clinical trials, for example, to probe for the treatment effects on human microbiome (2–6), the treatment effects on human health or disease (2, 4), and the roles of the microbiome on human health or disease (1, 2, 4, 7–14). It is also common in a clinical trial to trace disease status over a lengthy period to survey the sequential progress of disease or recovery for different treatment groups (e.g., treatment versus placebo, new treatment versus old treatment), not restricted to the disease status at a single specific time point (1, 2, 9, 12). Hence, disease responses are often available in the form of survival (i.e., time-to-event) responses that are stratified by different treatment groups.

**FIG 1:**
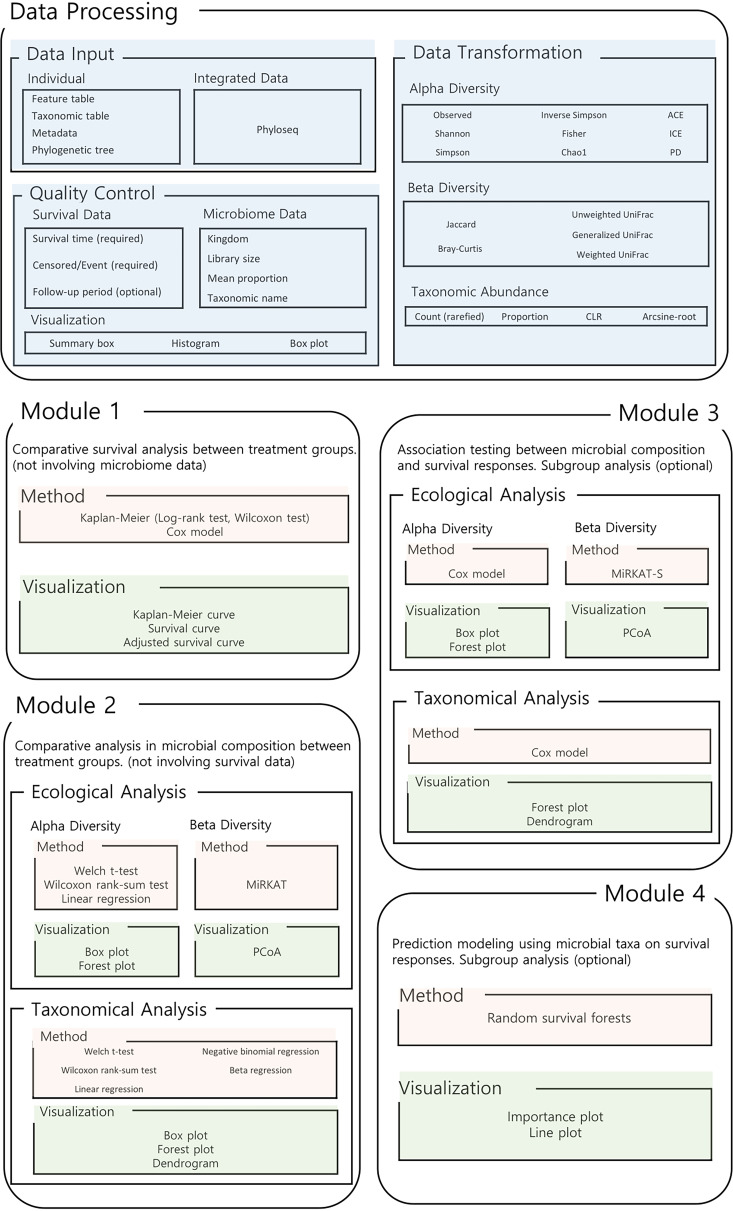
Overall workflow. MiSurv consists of a data processing module and its following four data analytic modules.

We therefore introduce here an integrative web cloud platform, called MiSurv, for microbiome data analysis with survival responses. MiSurv is a user-friendly step-by-step analytic pipeline like our previous web cloud platform, MiCloud (36). However, MiSurv is designed to deal with survival responses while MiCloud (36) is for continuous or binary responses. MiSurv performs microbiome data processing, analytic and graphical procedures comprehensively. MiSurv consists of a data processing module and its following four data analytic modules. Module 1: Comparative survival analysis between treatment groups. Module 2: Comparative analysis in microbial composition between treatment groups. Module 3: Association testing between microbial composition and survival responses. Module 4: Prediction modeling using microbial taxa on survival responses (Fig. 1). MiSurv is freely available on the web server (http://misurv.micloud.kr) or can alternatively run on the user’s local computer (https://github.com/wg99526/MiSurvGit).

In the following Results section, we delineate all the details for each module, while demonstrating them step-by-step through an example trial on the effects of antibiotic use on the survival rate against type 1 diabetes (T1D) onset (i.e., time-to-T1D onset) and gut microbiome composition, respectively, as well as the roles of the gut microbiome on the survival rate against T1D onset (4) (see Real data application). In the Discussion section, we summarize the results and finish with concluding remarks. Finally, in Materials and Methods, we describe the underlying machinery and web architecture of MiSurv.

## RESULTS

### Data processing: data input.

As in MiCloud (36), users need to upload four necessary data components: a feature table (i.e., count table for operational taxonomic units [OTUs] or amplicon sequence variants [ASVs]), a taxonomic table (i.e., taxonomic assignments at seven taxonomic levels, kingdom, phylum, class, order, family, genus, species), metadata (i.e., survival time, censored/event, demographics, etc. for the study subjects) and a phylogenetic tree (i.e., a rooted phylogenetic tree relating features [OTUs or ASVs]) (Fig. 1). Users can upload them as separate files or in a single unified format, called phyloseq (37) (Fig. 1).

**(i) Real data application.** We used the gut microbiome data for the study of antibiotic-induced acceleration of T1D (4). T1D is an autoimmune disease which is increasing in incidence globally with decreasing age of onset. To survey if the antibiotic use (i.e., a pulsed macrolide antibiotic [known as tylosin] treatment) accelerates T1D onset through gut microbiome perturbation, Zhang et al. conducted a randomized clinical trial using non-obese diabetic mice. Here, we use these data to demonstrate the use of MiSurv step-by-step. We described the raw sequence data repository and processing procedures in Materials and Methods. The final processed data (4) are also stored as example data in the phyloseq (37) format and also as four individual data files on these data processing module.

At this module, we uploaded these data clicking the “Upload” button.

### Data processing: quality control.

MiSurv performs quality controls for the survival data in follow-up period (Fig. 1). For this, users first need to select a variable for survival time (i.e., follow-up time) and a variable for censored or event (i.e., a binary indicator for censored [0] or event [1]). Then, users can adjust the follow-up period (start time and end time) of interest using a slide bar (or leave it as it is to survey the entire follow-up period just as given in the data), where the censored/event indicator is also automatically adjusted to be suited to the selected follow-up period. The rationale behind this quality control user option is that investigators often (but not always) set up a very long period of follow-up at a design stage because they do not exactly know how long they need to follow up in advance. A very long period of follow-up also tends to result in no disparity in survival rate between treatment groups because treatment effects tend to disappear (and/or converge to the same level) at a very late time in the end.

As in MiCloud (36), MiSurv also performs quality controls for the microbiome data in four criteria: kingdom (i.e., a kingdom of interest, default: Bacteria); library size (i.e., a minimum total read count for the study subjects to have, default: 3,000); mean proportion (i.e., a minimum mean proportion for the features [OTUs or ASVs] to have, default: 0.002%); taxonomic names (i.e., erroneous taxonomic names to be removed in the taxonomic table) (Fig. 1). MiSurv visualizes the microbiome data before and after quality controls using summary boxes, histograms and box plots. The microbiome data before and after quality controls can also be downloaded.

**FIG 2:**
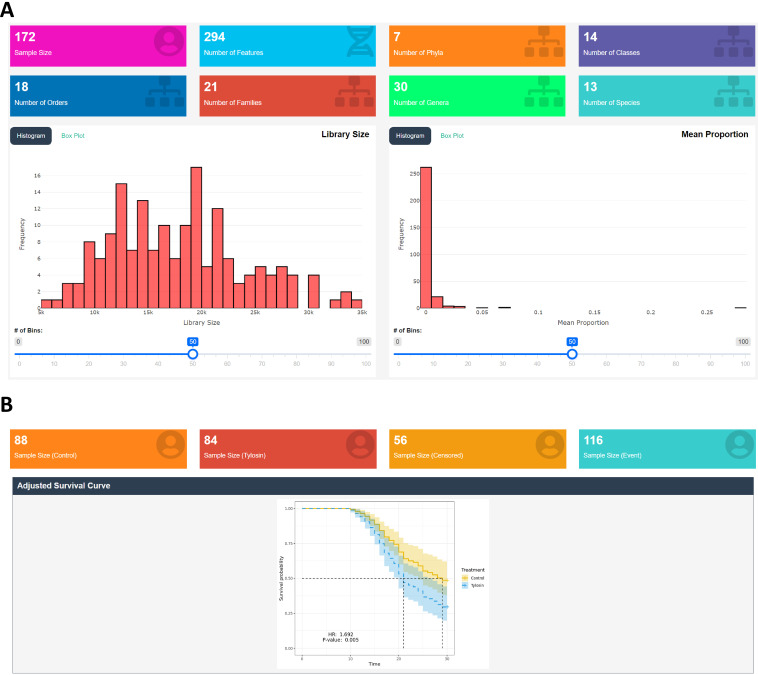
The screenshot after the quality controls using Data Processing (A) and the screenshot after performing comparative survival analysis between the control and tylosin groups adjusting for sex using Module 1 (B). *HR represents the estimated hazard ratio (i.e., the hazard rate for the tylosin group divided by the hazard rate for the control group).

**(i) Real data application.** We selected “T1Dweek” as the variable for the follow-up time, and “T1D” as the binary indicator for censored (0) or event (1), where the censored (0) represents T1D free and the event (1) represents T1D onset. We did not change the follow-up period as we wanted to survey the entire follow-up period just as given in the data. For the microbiome data, we applied the default quality control settings; as such, 294 features, 7 phyla, 14 classes, 18 orders, 21 families, 30 genera and 13 species for 172 subjects were finally retained (Fig. 2A).

### Data processing: data transformation.

As in MiCloud (36), for the ecological analysis, MiSurv calculates (1) nine alpha-diversity indices: Observed, Shannon (38), Simpson (39), Inverse Simpson (39), Fisher (40), Chao1 (41), abundance-based coverage estimator (ACE) (42), incidence-based coverage estimator (ICE) (43), and phylogenetic diversity (PD) (44); and (2) five beta-diversity indices: Jaccard dissimilarity (45), Bray-Curtis dissimilarity (46), Unweighted UniFrac distance (47), Generalized UniFrac distance (48), and Weighted UniFrac distance (49) (Fig. 1). For the taxonomical analysis, MiSurv transforms the original count data into four data forms: (i) count (rarefied) (50), (ii) proportion, (iii) centered log ratio (CLR) (51), and (iv) arcsine-root for each taxonomic rank (phylum, class, order, family, genus, species). While there is no consensus on which data format works best, the most popular microbiome data transformation method is the CLR transformation to deal with the compositionality of the data (51). Hence, we set CLR as the default for all following taxonomical analysis modules. All the calculated alpha-and beta-diversity indices as well as all the original and transformed taxonomic abundance data can also be downloaded.

**(i) Real data application.** We simply clicked the “Run” button to calculate the alpha- and beta-diversity indices, and transform the original count data into the four different data forms.

### Module 1: comparative survival analysis between treatment groups (not involving microbiome data).

Module 1 is for some basic exploratory data analysis, in which the microbiome data are not involved, to compare the survival rates between two treatment groups (e.g., treatment versus placebo, or new treatment versus old treatment), coded as 0 for the reference group and 1 for the comparison group (Fig. 1). For univariate analysis, the Kaplan-Meier analysis (default) (52) with the log-rank test (default) (53) or the Wilcoxon test (54), or the Cox model (55) can be employed. For covariate-adjusted analysis, only the Cox model (55) can be employed. The Kaplan-Meier analysis (52) and the Cox model (55) are the two most commonly used survival analysis methods. MiSurv visualizes the results using the Kaplan-Meier or covariate-adjusted survival curves. MiSurv also visualizes the sample sizes for different treatment groups and the sample sizes for the censored and events using summary boxes.

**(i) Real data application.** We selected “Antibiotics” as the treatment variable, where “Control” represents the group of normal controls and “Tylosin” represents the group of the subjects treated by a macrolide antibiotic, called tylosin (4). We selected “Sex” as a covariate, and then employed the Cox model (55) to perform covariate-adjusted analysis. We found a significant difference in survival rate between the control and tylosin groups (*P*-value: 0.005), while estimating that the hazard rate is greater for the tylosin group than the control group (hazard ratio [HR]: 1.692 > 1) (Fig. 2B). This indicates that the subjects treated by tylosin progresses to T1D onset more rapidly than the normal controls. The sample sizes for the control and tylosin groups were 88 and 84, respectively, while the sample sizes for the censored and events were 56 and 116, respectively (Fig. 2B).

### Module 2: comparative analysis in microbial composition between treatment groups (not involving survival data).

Module 2 is for other basic exploratory data analysis, in which the survival data are not involved, to compare the microbial composition between two treatment groups (e.g., treatment versus placebo, or new treatment versus old treatment), coded as 0 for the reference group and 1 for the comparison group (Fig. 1). MiSurv performs such comparative analysis with respect to alpha-diversity, beta-diversity and taxonomic abundance as in MiCloud (36). For the alpha-diversity analysis, the Welch *t* test or the Wilcoxon rank-sum test (default) (56) with box plots can be employed for univariate analysis, while the linear regression model with forest plots can be employed for covariate-adjusted analysis. For the beta-diversity analysis, MiRKAT (26, 27) can be employed for both univariate and covariate-adjusted analyses, yet only the principal coordinate analysis (PCoA) plots (57) are available to visualize the disparity between treatment groups in a univariate trend not reflecting covariate adjustments. For the taxonomic differential abundance analysis, four different data forms of the CLR (default) (51), count, proportion and arcsine-root are available. For the CLR (default) and arcsine-root, the Welch *t* test or the Wilcoxon rank-sum test (default) (56) with box plots can be employed for univariate analysis, while the linear regression model with forest plots can be employed for covariate-adjusted analysis. For the count, the Welch *t* test or the Wilcoxon rank-sum test (default) (56) using rarefied counts (50) with box plots can be employed for univariate analysis, while the negative binomial regression model using original counts, including the library size as an off-set variable with forest plots can be employed for covariate-adjusted analysis. For the proportion, the Welch *t* test or the Wilcoxon rank-sum test (default) (56) with box plots can be employed for univariate analysis, while the beta regression model with forest plots can be employed for covariate-adjusted analysis. Users can survey from phylum to genus (default) (e.g., for 16S rRNA sequencing) or from phylum to species (e.g., for shotgun metagenomics). For any taxonomic differential abundance analysis, MiSurv applies the Benjamini-Hochberg (BH) procedures (58) to each taxonomic rank to control for the false discovery rate (FDR) at 5%. MiSurv also visualizes the hierarchical taxonomic discovery status using a dendrogram.

**FIG 3:**
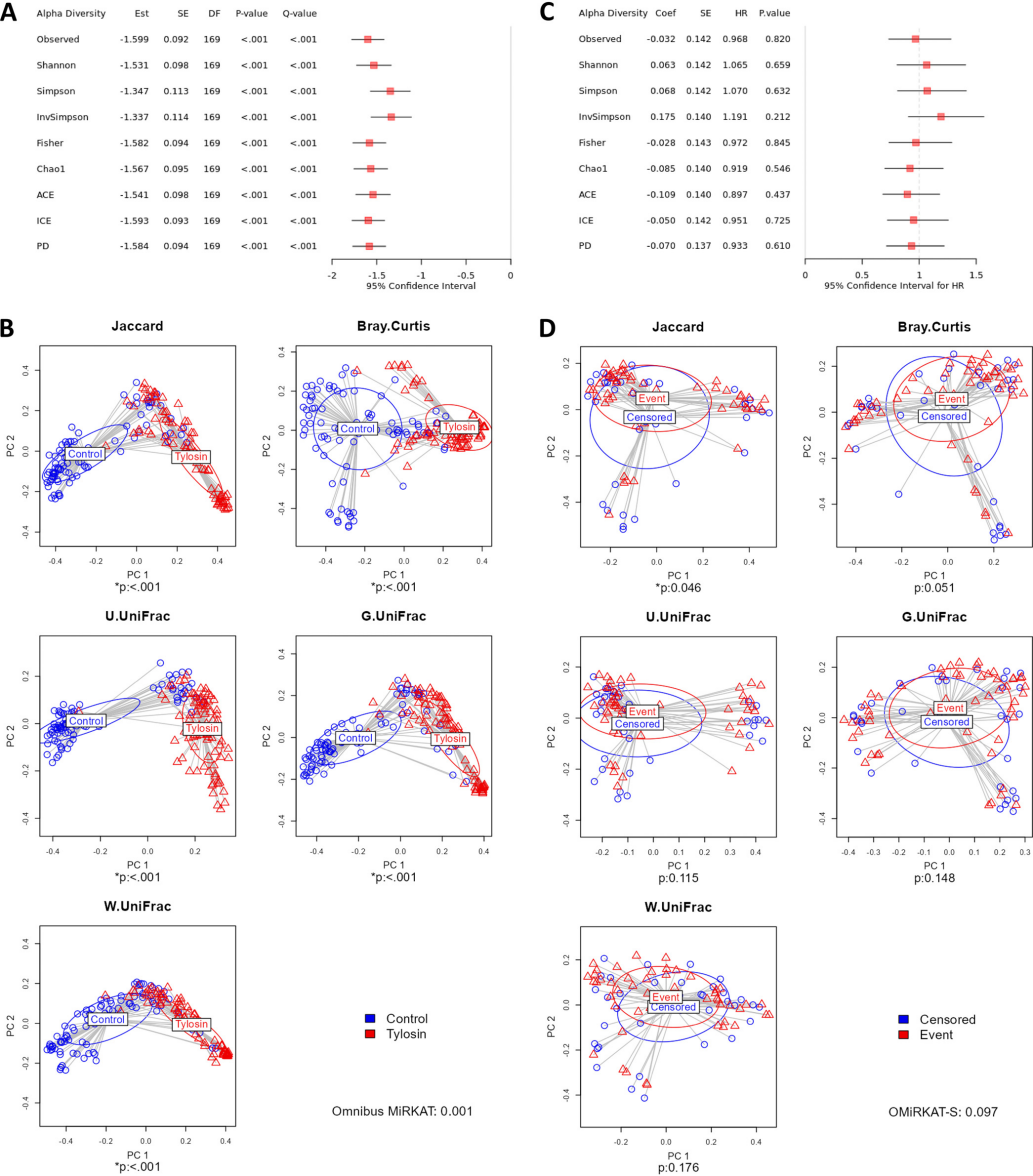
The results from alpha-diversity (A) and beta-diversity (B) analyses to compare the microbial compositions between the control and tylosin groups adjusting for sex using Module 2, and the results from alpha-diversity (C) and beta-diversity (D) analyses to test the association with survival responses for the control group adjusting for sex using Module 3.

**FIG 4:**
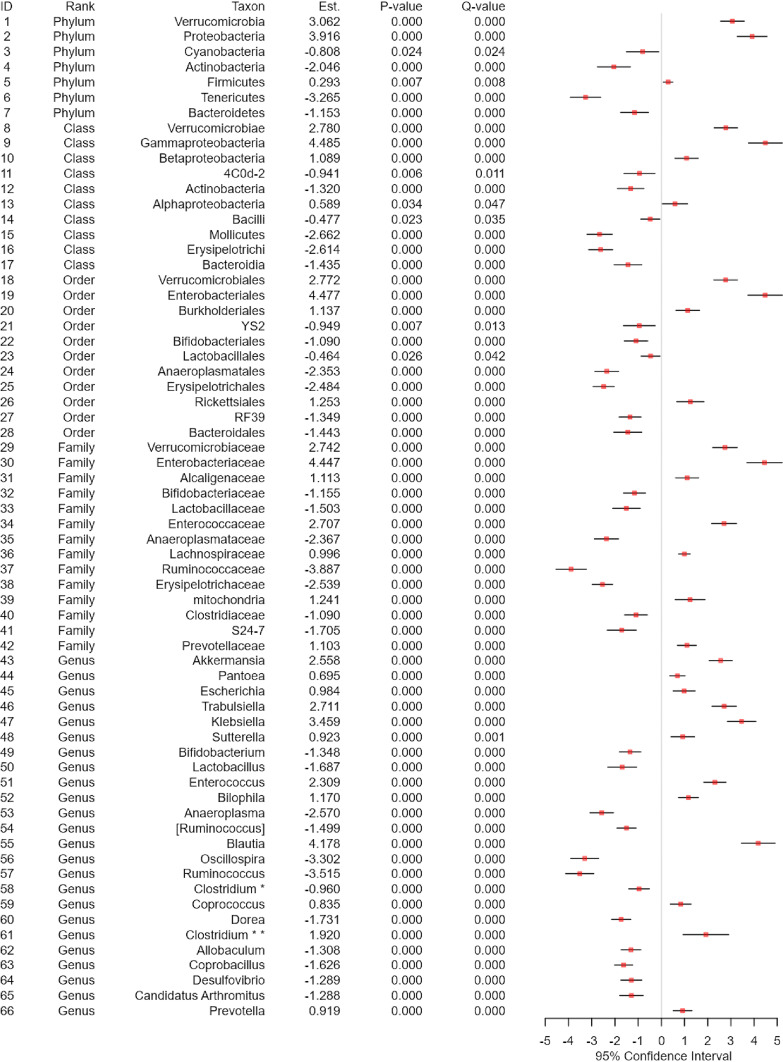
The forest plot for taxonomic differential abundance analyses adjusting for sex using Module 2.

**FIG 5:**
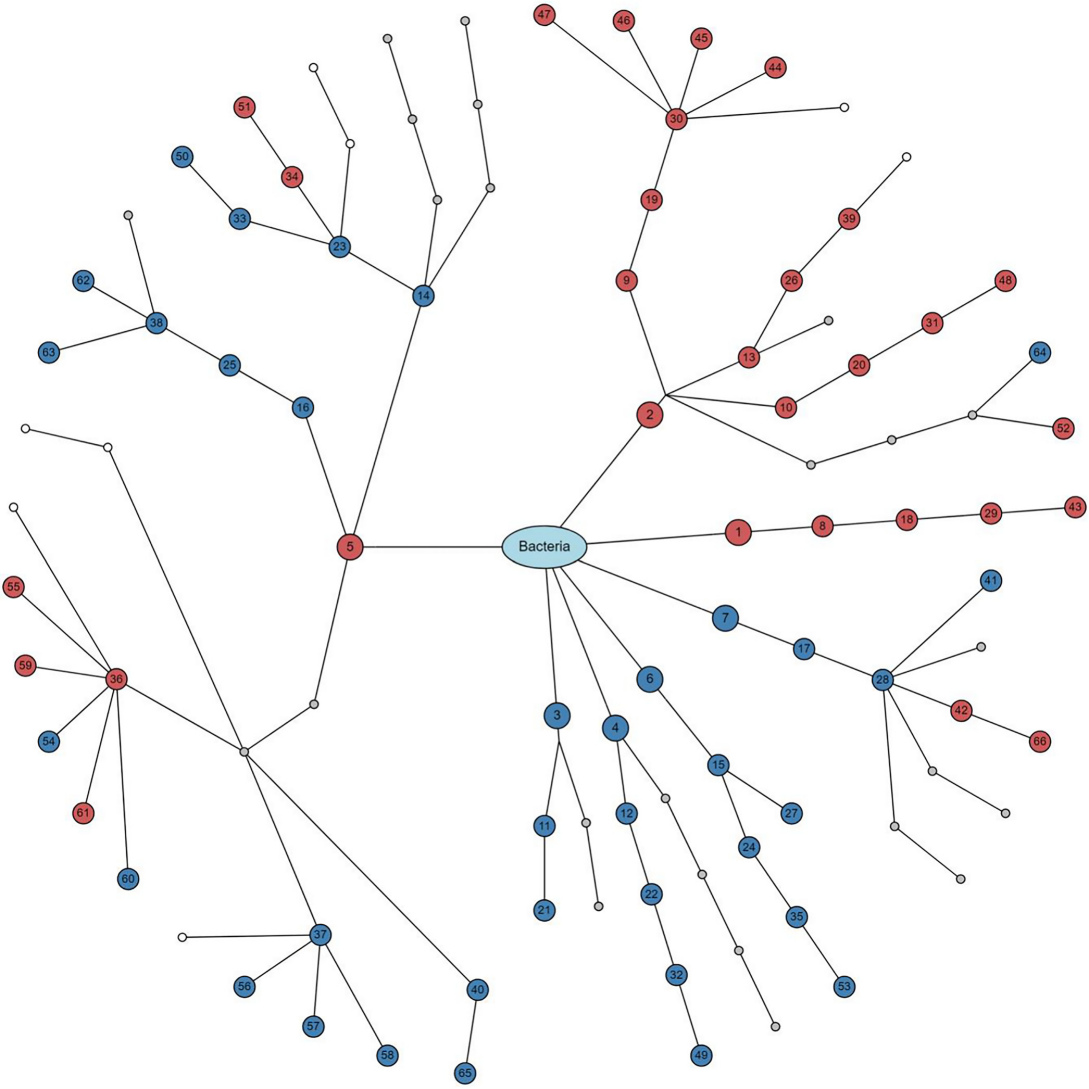
The dendrogram for taxonomic differential abundance analyses adjusting for sex using Module 2. The numbers in circles are the taxon IDs in the forest plot (Fig. 4). Red: positive association. Blue: negative association. Gray: non-significance. White: no association testing for non-taxonomic annotation.

**(i) Real data application.** We selected “Antibiotics” as the treatment variable, where “Control” represents the group of normal controls and “Tylosin” represents the group of the subjects treated by a macrolide antibiotic, called tylosin (4). We selected “Sex” as a covariate, and then performed covariate-adjusted analyses using the linear regression model for the alpha-diversity analysis (Fig. 3A), using MiRKAT (26, 27) for the beta-diversity analysis (Fig. 3B), and using the linear regression model with the CLR (default) (51) transformed data for the taxonomic differential abundance analysis (Fig. 4 and 5). Then, we found a significant difference in alpha-diversity between the control and tylosin groups for all the nine alpha-diversity indices (Fig. 3A), estimating that the alpha-diversity is smaller for the tylosin group than the control group for all nine alpha-diversity indices (Fig. 3A). We also found a significant difference in beta-diversity between the control and tylosin groups for all five beta-diversity indices (Fig. 3B). Finally, we discovered 66 taxa (from phylum to genus) that have significant disparity in relative abundance between the control and tylosin groups (Fig. 4 and 5).

### Module 3: association testing between microbial composition and survival responses.

Module 3 involves both microbiome data and survival data and performs association testing between microbial composition and survival responses with respect to alpha-diversity, beta-diversity and taxonomic abundance. Each submodule for the alpha-diversity, beta-diversity or taxonomic abundance begins with subsetting the data for subgroup analysis. That is, users can select a subgroup of interest using a category of a nominal variable (usually the treatment variable) to perform subgroup analysis. If they select a subgroup, only the subjects in that subgroup are retained in the following analysis. Otherwise, all the subjects just as given in the data are retained. The rationale behind this subgroup analysis user option is that the effects of microbiome on survival responses are usually different between treatment groups, indicating an interaction between microbiome and treatment.

For the alpha-diversity analysis, the Cox model (55) with forest plots can be employed for both univariate and covariate-adjusted analyses. For the beta-diversity analysis, MiRKAT-S (27, 59, 60) with PCoA plots (57) can be employed for both univariate and covariate-adjusted analyses. Here, again, the PCoA (57) plots do not reflect covariate adjustments and display the disparity in microbiome composition between the censored and events only at the end of the follow-up. For the taxonomic association analysis, four different data forms of the CLR (default) (51), count (rarefied) (50), proportion and arcsine-root are available. Then, the Cox model (55) with forest plots can be employed for both univariate and covariate-adjusted analyses. As in Module 2, users can survey from phylum to genus (default) (e.g., for 16S rRNA sequencing) or from phylum to species (e.g., for shotgun metagenomics). MiSurv applies the BH procedures (58) to each taxonomic rank to control for the FDR at 5%. MiSurv also visualizes the hierarchical taxonomic discovery status using a dendrogram.

**FIG 6:**
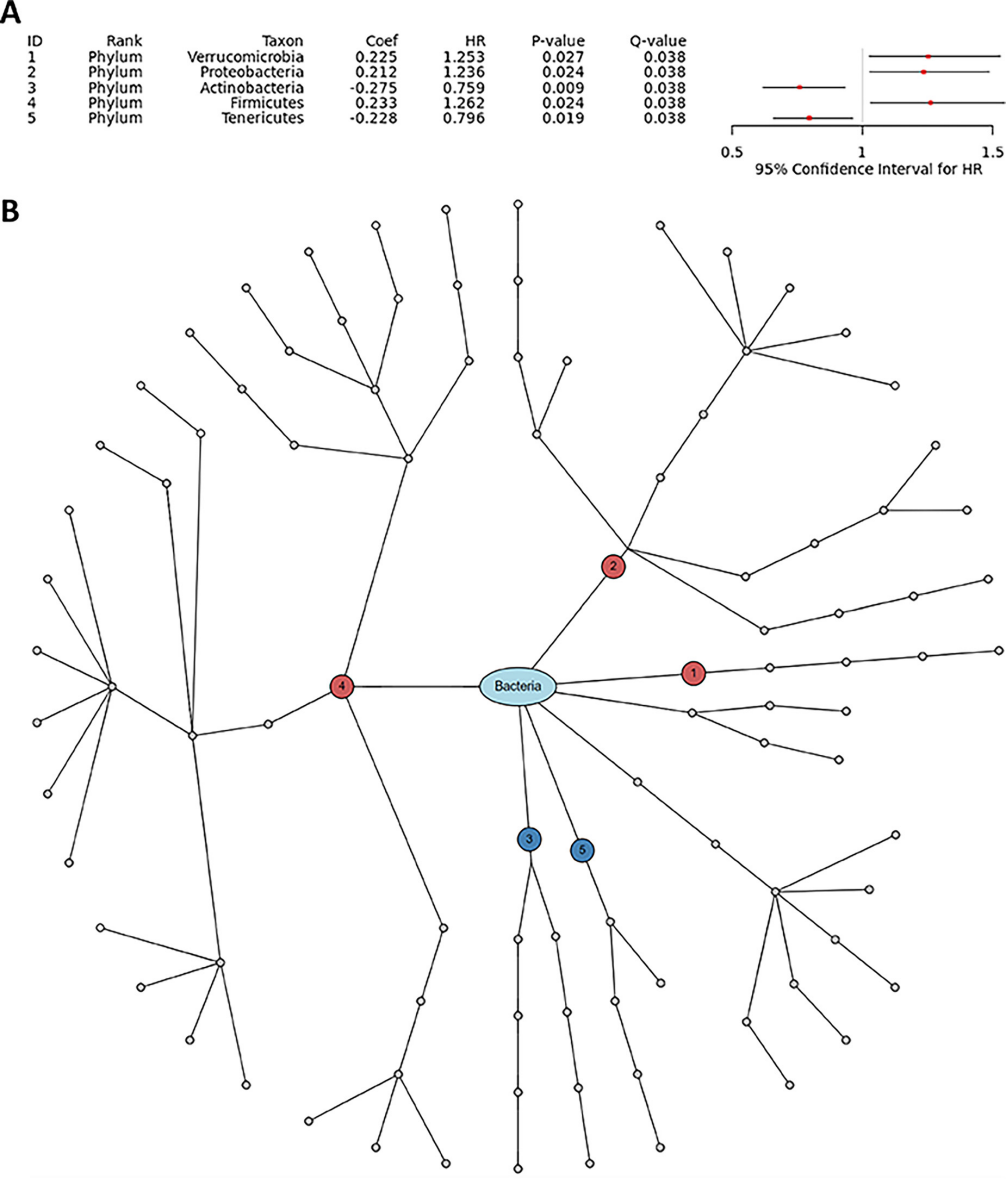
The forest plot (A) and dendrogram (B) for taxonomic association analyses between each taxon and survival responses adjusting for sex using Module 3. The numbers in circles (Fig. 6B) are the taxon IDs in the forest plot (Fig. 6A). Red: positive association. Blue: negative association. Gray: non-significance. White: no association testing for non-taxonomic annotation. *HR represents the estimated hazard ratio (i.e., the relative increase in hazard rate with one unit increase in the CLR transformed relative abundance).

**(i) Real data application.** We performed subgroup analysis while selecting the control group in the treatment variable as the subgroup of interest; as such, only the normal controls (excluding tylosin subjects) were retained in the following analyses. We selected “Sex” as a covariate to perform covariate-adjusted analyses. For the alpha-diversity analysis, we did not find any significant association between alpha-diversity and survival responses for any alpha-diversity index (Fig. 3C). For the beta-diversity analysis, we found a significant association between beta-diversity and survival responses for the Jaccard dissimilarity (*P* value: 0.046) (Fig. 3D). Finally, we discovered 5 phyla (Verrucomicrobia, Proteobacteria, Actinobacteria, Firmicutes, Tenericutes) that have significant association with the survival responses (Fig. 6). We estimated that the hazard rate is greater with increasing CLR transformed relative abundance for Verrucomicrobia (HR: 1.253 > 1), Proteobacteria (HR: 1.236 > 1) and Firmicutes (HR: 1.262 > 1), indicating the subjects that have more Verrucomicrobia, Proteobacteria and Firmicutes in their gut progresses to T1D onset more rapidly. On the contrary, we estimated that the hazard rate is smaller with increasing CLR transformed relative abundance for Actinobacteria (HR: 0.759 < 1) and Tenericutes (HR: 0.796 < 1), indicating the subjects that have less Actinobacteria and Tenericutes in their gut progress to T1D onset more slowly (Fig. 6).

### Module 4: prediction modeling using microbial taxa on survival responses.

Module 4 involves both microbiome data and survival data, and performs prediction modeling using microbial taxa at the taxonomic ranks of phylum, class, order, family, genus and species, respectively, on survival responses. As in Module 3, users can select a subgroup of interest using a category of a nominal variable (usually the treatment variable) to perform subgroup analysis. As in Module 3, users first need to select a data form among CLR (default) (51), count (rarefied) (50), proportion and arcsine-root. Then, they can survey from phylum to genus (default) (e.g., for 16S rRNA sequencing) or from phylum to species (e.g., for shotgun metagenomics). For prediction modeling, the random survival forests (61, 62) can be employed. The random survival forests (61, 62) is an ensemble learning method that averages the predictions on survival rates resulting from a multitude of decision trees constructed by randomly selected inputs. Hence, the random survival forests can robustly suit various linear or non-linear patterns while decorrelating taxa and adapting to various sparsity levels. Users can select the number of trees (5,000 [default] or 10,000) to be ensembled. Users can also select the number of taxa at each taxonomic rank to be displayed on the variable importance plot (61, 62) that ranks taxa in the amount of increase in the residual sum of squares. MiSurv also visualizes the out-of-bag error rates by the number of trees to be ensembled.

**FIG 7:**
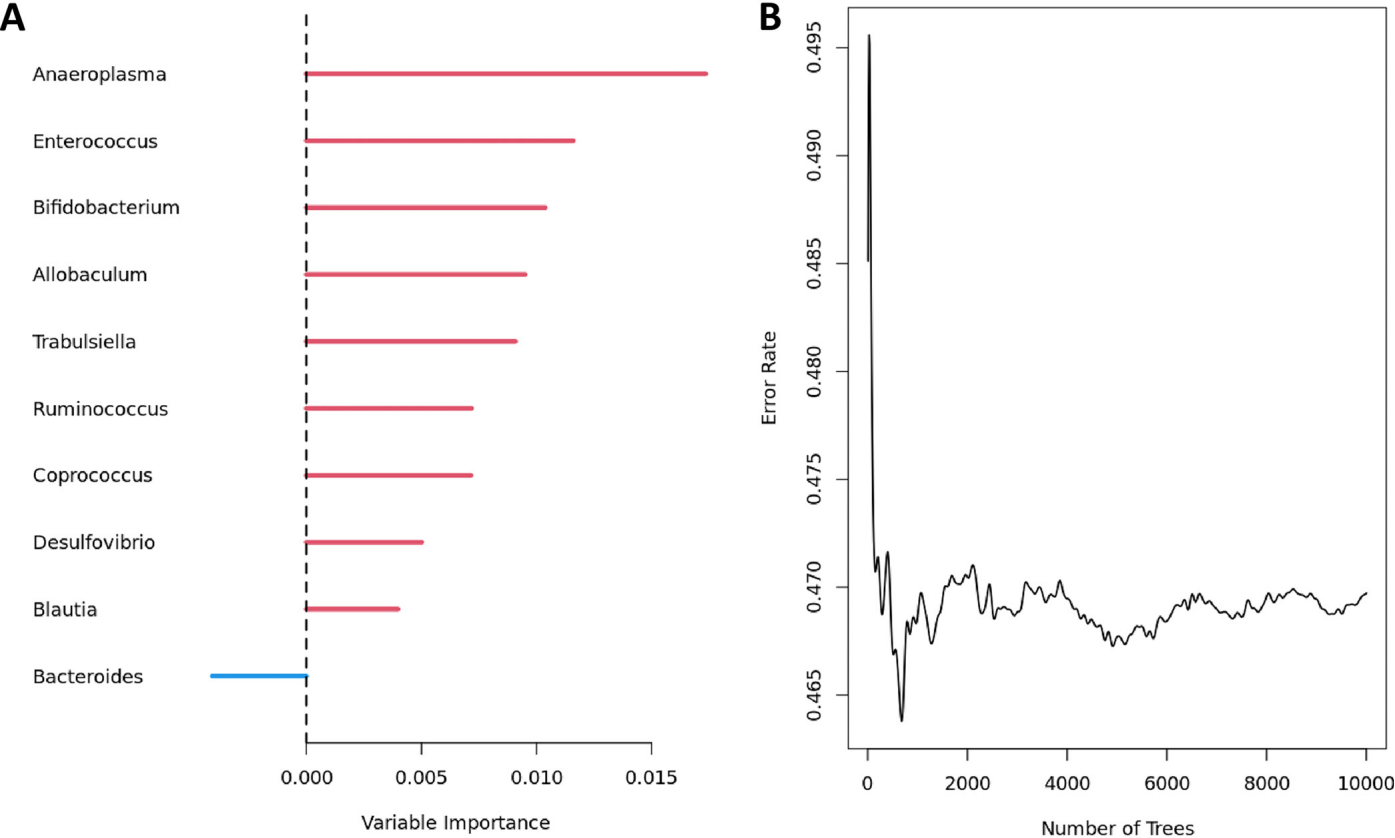
The importance plot (A) and error rates (B) for prediction modeling using genera on survival responses using Module 4.

**(i) Real data application.** We performed subgroup analysis while selecting the control group in the treatment variable as the subgroup of interest; as such, only the normal controls (excluding tylosin subjects) were retained in the following analysis. We selected 10,000 as the number of trees to be ensembled and 10 as the number of taxa at each taxonomic rank to be displayed on the variable importance plot. We listed top 10 genera that have the most influential predictions on the survival rates in (Fig. 7A). We also observed that the out-of-bag error rates well converge by 10,000 of trees ensembled in (Fig. 7B).

## DISCUSSION

In this paper, we introduced an integrative web cloud platform, MiSurv, for user-friendly step-by-step microbiome data analysis with survival responses. MiSurv performs extended data processing, analytic and graphical procedures to efficiently deal with microbiome data with survival responses. We described in detail a data processing module and its following four data analytic modules of MiSurv along with the example gut microbiome trial that involves a treatment (antibiotics) variable and survival responses on T1D onset.

In the Data Processing module, we added the quality control to adjust the follow-up period because investigators often set up a very long period of follow-up at the stage of experimental design just to survey the disease progression sufficiently. Though, it is usually not the one intended in a later data analysis. Module 1 and Module 2 perform some basic exploratory data analysis, not involving microbiome and survival data, respectively, for comparative survival analysis between treatment groups and comparative analysis in microbial composition between treatment groups, respectively. On the contrary, Module 3 and 4 link the microbial composition to survival responses with respect to association testing and prediction modeling, respectively. At the beginning of Module 3 and 4, we added the subgroup analysis user option because it is often assumed that the effects of microbiome on survival responses are different between treatment groups, which indicates an interaction between microbiome and treatment, which in turn indicates the need for subgroup analysis. We believe that not all the facilities in MiSurv are just ours. Indeed, they are mostly based on other researchers’ methodologies; hence, we devoted to adding all related references to each of the data processing and analytic steps on our web platform.

In summary, MiSurv is easy to use and provides comprehensive facilities for microbiome data processing and downstream data analysis with survival responses. MiSurv can serve as a useful tool in the rapidly growing field of human microbiome for numerous trials to study the treatment effects on survival responses, the treatment effects on human microbiome, and the roles of the microbiome on survival responses.

## MATERIALS AND METHODS

### Overall architecture.

We made all the user interfaces and server functions using R Shiny (https://shiny.rstudio.com). The computer server currently has Intel Core i7-12700T processor (12 cores,1.40 to 4.70 GHz) and 36 GB DDR4 memory and runs on the Ubuntu 20.04 server. We will improve the specification if it gets more popular and busier. A web server, in theory, is any software that can interact with another entity over a network. The overall architecture of our web server follows a typical client-server model, deploying it through two layers, (1) ShinyProxy (https://www.shinyproxy.io) to maintain our service stable by means of the docker and container, and (2) Apache2 (https://httpd.apache.org) to provide easier access to our service by means of the domain name. Our web server currently supports up to 10 simultaneous connections. We also host our platform through GitHub (https://github.com) repository for the users who wish to run it on their local computer.

### Underlying machinery.

We wrote all the data processing, analytic and graphical procedures in R language [Key Resource Table]. MiSurv calculates the alpha-diversity indices (38–44) using “estimate_richness” in the “phyloseq” package and “pd” function in the “picante” package. MiSurv calculates the beta-diversity indices (45–49) using the “dist” function in the “proxy” package, the “bcdist” function in the “ecodist” package, and the “GUniFrac” in the “GUniFrac” package. MiSurv conducts the CLR (51) transformation using the “clr” function in the “compositions” package and the rarefaction (50) using the “rarefy_even_depth” in the “phyloseq” package. MiSurv performs the Kaplan-Meier analysis (52) with the log-rank test (53) and Wilcoxon test (54) using the “Surv” and “survdiff” functions in the “survival” package. MiSurv fits the Cox model (55) using the “coxph” function in the “survival” package. MiSurv performs MiRKAT (26, 27) and MiRKAT-S (27, 59, 60) with PCoA plots (57) using the “D2K,” “MiRKAT,” and “MiRKATS” functions in the “MiRKAT” package and the “pcoa” function in the “ape” package. MiSurv performs the Welch *t* test using the “t.test” function in the “stats” package and the Wilcoxon rank-sum test (56) using the “wilcox.test” function in the “stats” packages. MiSurv fits the linear regression model using the “lm” and “glm” function in the “stats” package, the negative binomial regression model using the “glm.nb” function in the “MASS” package, and the beta regression model using the “betareg” function in the “betareg” package. MiSurv fits the random survival forests (61, 62) using the “rfsrc” function in the “randomForestSRC” package.

### Raw sequence data processing procedures.

Zhang et al. collected fecal samples from non-obese diabetic mice from Jackson Laboratory (4). Then, they extracted intestinal microbiota DNA using the PowerLyzer PowerSoil DNA isolation kit (MoBio, Carlsbad, CA) and the PowerSoil-htp 96-Well Soil DNA isolation kit (MoBio). Then, they acquired the amplicon library of V4 regions of the 16S rRNA genes through triplicate PCR with barcoded fusion primers, quantification with the Qubbit 2.0 Fluorometer (Life Technologies, Carlsbad, CA). and combination of each DNA sample at equal concentrations as in (2). Then, they sequenced the library using Illumina MiSeq 2 × 150 bp paired end platform (Illumina, San Diego, CA).

We used QIIME 2.0 [16] to obtain the feature table, taxonomic annotations, and phylogenetic tree. We retained only the reads with >75% of the original length while removing the reads with more than three consecutive low-quality bases (Phred score < 20). We constructed (1) the feature table using the 97% sequence similarity through open reference piking based on the Greengenes database (63), (2) the taxonomic annotations using the RDP classifier while removing chimeras using ChimeraSlayer (64), and (3) the phylogenetic tree using FastTree (65).

### Data and code availability.

We used public microbiome data for our real data applications (4). The raw sequence data are deposited in QIITA (https://qiita.ucsd.edu) with the ID number 10508 (https://qiita.ucsd.edu/study/description/10508). All the final processed data are also stored in the Data Processing: Data Input module on the web server (http://misurv.micloud.kr) and also in the Data folder on the GitHub page (https://github.com/wg99526/MiSurvGit) with the for the “phyloseq” (37) object (see the file named “biom.Rdata”) and also with four individual files (see the files named “otu.tab.txt,” “sam.dat.txt,” “tax.tab.txt,” and “tree.tre”).

MiSurv can be run through our web server (http://misurv.micloud.kr) or on the user’s local computer through the GitHub repository (https://github.com/wg99526/MiSurvGit). Either of these platforms comes with a detailed software manual on implementation, example data (4), required or optional inputs or settings, numeric or graphical outputs, related references, and so forth.
